# Carbon Nanotube Migration in a Compatibilized Blend System, Leading to Kinetically Induced Enhancement in Electrical Conductivity and Mechanical Properties

**DOI:** 10.3390/nano13061039

**Published:** 2023-03-14

**Authors:** Lilian Azubuike, Jun Wang, Uttandaraman Sundararaj

**Affiliations:** 1 Department of Chemical and Petroleum Engineering, University of Calgary, 2500 University Drive NW, Calgary, AB T2N 1N4, Canada; 2 Advanced Materials Thrust, The Hong Kong University of Science and Technology (Guangzhou), Guangzhou 511453, China; 3 Department of Chemical and Biological Engineering, The Hong Kong University of Science and Technology, Hong Kong 999077, China

**Keywords:** kinetics, interface, high-density polyethylene, poly phenylene ether/oxide, carbon nanotube, SEBS, migration, compatibilization, electrical conductivity, tensile properties, structure–property relationship

## Abstract

Kinetic factors that facilitate carbon nanotube (CNT) migration in a polymer blend from a high-density polyethylene (HDPE) phase to a poly (p-phenylene ether) (PPE) phase were studied, with the objective to induce CNT migration and localization at the interface. Herein, a CNT filler was pre-localized in an HDPE polymer and then blended with PPE at different blend compositions of 20:80, 40:60, 60:40, and 80:20 of PPE/HDPE at a constant filler concentration of 1 wt%. The level of CNT migration was studied at different mixing times of 5 and 10 min. The electrical conductivity initially increased by 2–3 orders of magnitude, with an increase in the PPE content up to 40%, and then it decreased significantly by up to 12 orders of magnitude at high PPE content up to 100%. We determined that the extent of migration was related to the difference in the melt viscosity between the constituent polymers. A triblock copolymer styrene-ethylene/butylene-styrene (SEBS) was used to improve the blend miscibility, and 2 wt% copolymer was found to be the optimum concentration for the electrical properties for the two blend compositions of 20:80 and 80:20 of PPE/HDPE, at a constant filler concentration of 1 wt%. The introduction of the SEBS triblock copolymer significantly increased the conductivity almost by almost four orders of magnitude for PPE/HDPE/80:20 composites with 1 wt% CNT and 2 wt% SEBS compared to the uncompatibilized blend nanocomposite. The mechanical strength of the compatibilized blend nanocomposites was found to be higher than the unfilled compatibilized blend (i.e., without CNT), uncompatibilized blend nanocomposites, and the pristine blend, illustrating the synergistic effect of adding nanofillers and a compatibilizer. SEM and TEM microstructures were used to interpret the structure–property relationships of these polymer blend nanocomposites.

## 1. Introduction

Polymer blending is a creative pathway to combine the strengths of different materials and help improve the deficient properties of a particular material [1,2,3]. Due to the macromolecular nature of polymers, they tend to have low entropy of mixing; hence, they show phase separation and poor properties. This is mostly because of long chains of two or more polymers that are not able to adhere together during processing at a molecular level [4]. The widely applied strategy to improving the structural properties of an immiscible polymer blend is addition of block copolymers, which has been shown in many research studies to refine and help stabilize the morphology of immiscible blends [5]. The inherent properties of constituent polymers in a blend can be used to systematically manipulate the final composite by tailoring the interphase of the multiphase system, a strategy that has shown to improve the interfacial properties, especially in the polymer blending process. In addition, the introduction of carbon nanotube filler (NC7000), an outstanding nanofiller that has many applications [6], contributes also to the final blend properties as each constituent polymer has a different level of affinity towards this nanofiller; hence, its final localization can be determined either by thermodynamic and kinetic [7] properties and, at same time, affect the final blend morphology. In terms of the thermodynamics, the interfacial relationship between different components in the blend, helps to predict the nanofiller localization, using the wettability parameter. The high interfacial tension during processing and poor interfacial interactions in the macromolecular state contributes to a poor composite performance [7,8,9,10,11]. While for kinetics, the general processing conditions, the viscosity mismatch of the individual polymers in the blend, determines the selective localization of the nanofiller in blend composites. In a dispersed morphology of an immiscible blend, the droplets are usually irregular, resulting in a weak interphase, thus, when force is applied on the material, a fracture results on the interphase between the phases [9,10,11,12,13]. Introducing nanomaterial into this morphological structure; a possible filler migration between polymer phases occurs, which impacts the domain size and the interfacial properties. Fiona and Esmail studied the effect of Janus silica nanoparticles on the rheological and mechanical properties of a droplet-matrix blend configuration of polystyrene/poly(methyl methacrylate), PS/PMMA, and reported that the Janus particle improved the processability of the blend by decreasing their viscosity and consequently increasing the tensile modulus as result of the localization of the particle at the blend interphase [14]. Some research also shows that in a dispersed morphology, the presence of the droplets results in an improvement in the blend elasticity at low frequencies caused by stresses as result of the shape relaxation of the droplets induced by interfacial tension between the phases [15,16,17,18,19]. On the other hand, in a co-continuous morphology, each individual polymer simultaneously contributes to the end use properties, especially in melt mixing. To achieve optimum properties in a co-continuous structure, it is important to synergistically control the filler migration and other processing conditions, focusing on the conditions that establish morphology stability [20]. In this microstructure, the rheological and interfacial interaction depends on the constitute phases, which usually exist in the intermediate compositions; hence, they can limit the target properties [21]. Subsequently, the addition of nanofillers in a co-continuous structure helps to stabilize the microstructure, such that the interfacially localized nanofillers reduce the interfacial tension in the polymer blend [22], enhance the continuity of the minor phase, and, at the same time, decrease the percolation threshold. Jianwen C. et al., in their work, achieved a low percolation threshold by controlling carbon nanotube (CNT) filler migration at the interphase of the PS/PMMA blend, through the balance of the π-π and the dipole–dipole interaction in co-continuous morphology [23]. Bose et al., on the other hand, employed a reactive modifier that enhanced the formation of the CNT network structure in a co-continuous structure of nylon6/acrylonitrile–butadiene–styrene (PA6/ABS); they reported a significant refinement of the morphological structure and a well uniformed dispersion of the modified nanofiller in the PA6 phase [24]. Furthermore, the filler movement in an immiscible blend can situate at the interphase or, on either phase, can be systematically tuned depending on the target application. Individual polymer properties contribute to the kinetics of the blend processing, specifically for high viscous polymers such as poly (p-phenylene ether) (PPE) that are relatively difficult to process and are blended with other polymers such as polystyrene, nylons, and polyolefins [23,24,25,26]. The high viscosity of this PPE polymer can be useful in controlling nanofillers migration due to the high shear rate caused by the phase viscosity; hence, in a given blend with PPE, it can be utilized to limit nanofillers migration and subsequently trap them at the interphase. Many research [27,28,29] works have studied PPE systems with other polymers such as nylon66 (PA66) to enhance their processability and, at the same time, tune CNT migration. Chang Jae Lee et al. showed different sequences of mixing (PPE/PS)/PA66/CNT blend nanocomposites, in which CNTs dispersion and localization were induced by PA66 that has a high affinity for the nanofillers; they inferred that CNTs migrated from an unfavorable PS/PPE phase to a more favorable PA66 phase in a “filler-transfer-induced dispersion” mechanism [29]. Moreover, the effect of CNT dispersion in a miscible PPE/PS blend, studied by Qiyan Zhang et al., attributed the melt viscosity in relation to the PS amount, in which an optimum balance in the breakage of CNT agglomerate between the intrusion of polymer(matrix) molecules into CNT agglomerates and disintegrates the agglomerates by shear stress, contributing to a better dispersion and subsequently increased electrical conductivity [30]. Bing Du et al. investigated the blend of SAN/PPE filled with a functionalized multi-walled carbon nanotube, in which a pristine multi-walled carbon nanotube (MWCNT) remained in the pre-localized styrene-acrylonitrile (SAN) phase, while the functionalized MWCNT migrated to the PPE phase due to the grafted PS on the nanofillers surface [31].

In this work, we investigate the extent of CNT migration, kinetically induced in a compatibilized PPE/HDPE (high-density polyethylene) polymer blend nanocomposite as it narrows the interphase through trapping the filler there. In details, the PPE/HDPE blend of different blend compositions are studied at different mixing times in the melt mixing process, adding to the high viscosity difference, which was employed to manipulate CNT migration. PPE polymer basically has vast engineering applications in automotive and construction industries, but it has a drawback in its usage due to its high viscosity in the melt state; hence, it is blended with other polymers to improve its processability. Herein, we selected polyolefin, a HDPE, because it is relatively cheap and has a good chemical resistance, and a widely used household polymer. However, the PPE/HDPE blend is highly immiscible, so in our study, we added a styrene–ethylene butylene–styrene (SEBS) triblock copolymer as a compatibilizer. Minho Lee et al. studied the effect of polyphthalamide (PA6T) as a compatibilizer in a PA66/PPE blend nanocomposites; they inferred that due to the high affinity of CNT for PA6T, it improved the filler dispersion and, subsequently, the electrical conductivity of the PA66/PA6T/PPE/CNT composites [30]. Our study is uniquely focused on improving the processing of PPE polymer by blending with the HDPE/CNT masterbatch, with the SEBS copolymer to improve and stabilize the different phase morphologies at different mixing times being examined.

## 2. Materials and Methods

The polymers used in this study are high viscosity PPE Noryl^TM^ (640-111) supplied by SABIC and HDPE Novapol^®^ TR-0740-U provided by Nova Chemicals. The SEBS triblock copolymer (A1535HU), with a 60 wt% styrene content, was provided by Kraton^TM^ Corporation. Multi-walled carbon nanotubes (MWCNTs) Nanocyl^TM^ NC 7000 were procured from Nanocyl S.A (Sambreville, Belgium) and were used as the nanofiller.

### 2.1. Blend Preparation

The PPE/HDPE blend nanocomposites were prepared in a two-step melt mixing process. The HDPE was mixed with 10 wt% CNT in a Process 11 mini extruder to make a masterbatch (MB). The extrusion was performed at 50 rpm speed and a temperature range of 140–200 °C and was subsequently fed into a pelletizer to make pellets 2–4 mm in size.

The HDPE-MB was then diluted using a pure HDPE polymer and then mixed with a PPE polymer, to obtain the PPE/HDPE/ 1 wt% CNT blend composition in the second step of mixing. The PPE/HDPE/CNT were processed at four different blend compositions, 20:80, 40:60, 60:40, and 80:20 of PPE/HDPE, simultaneously, in a mini batch mixer and an Alberta Polymer Asymmetric mini mixer (APAM) [32] at 260 °C and a 200 rpm speed. The mixing was done at two different mixing times of 5 and 10 min to study the extent of CNT migration from the pre-localized phase to the HDPE and the PPE phase. Furthermore, the blend compositions were scaled up using our internal mixer, Thermo Fisher Haake^TM^ Rheomix series 600^®^ with roller rotors, which are used predominately for thermoplastics polymers. The chamber size is 64 cm^3^ and it has a 70% filling capacity; this was used to make enough samples for mechanical test. There has been an extensive study [33,34] of the effect of the scale-up in morphology and, consequently, the properties. If the mixing equipment has enough similarity to the scaled-up equipment, then the morphology can be scaled up. Compatibilized composites were prepared at different concentrations of SEBS triblock copolymer, 1, 2, and 3 wt%, and the final properties compared with the uncompatilized composites. The sample concentrations are summarized in Table 1 below.

### 2.2. Sample Characterization and Testing

The phase morphology of all the blend nanocomposites was captured using SEM, Quanta FEG 250 VP-FESEM from FEI Company, Hillsboro, OR, USA. The backscattered and secondary electron images were acquired under high vacuum conditions with an accelerating voltage of 10 kV. Backscatter images were collected using a two-segment Si photodiode-type detector, while secondary electron images were acquired using an Everhart–Thornley detector. Prior to SEM, all the samples were cryo fractured using liquid nitrogen and then etched with chloroform to extract the PPE phase.

Transmission electron microscopy was carried out to study the CNT migration across the polymer phases and the impact of the copolymer. An ultrathin section of composites, approximately 50 nm, was cut using a Leica EM FC6 ultra microtome (Leica Bio systems^©^, Nussloch, Germany) after trimming the sample surface and then sectioning with a diamond knife under liquid nitrogen at −120 °C. The TEM image was then obtained using a Tecnai F20 TEM microscope at 200 KV, attached with an FEI eagle camera.

The CNT dispersion and distribution was visualized using optical microscopy (OM). The samples were first sectioned to a 1 μm thickness using the EM FC6 ultramicrotome under liquid nitrogen, and then the sections were placed under a microscope glass slide and viewed with an Olympus^®^ BX60 optical 176 microscope (Olympus Inc., Tokyo, Japan) connected to an Olympus DP80 camera.

To measure the polymers viscosities, a rheological evaluation was carried out using an Anton-Paar MCR 302 rheometer at 260 °C, using a 25 mm diameter parallel-plate geometry with gap size of 1 mm, under nitrogen to minimize the degradations of the polymers during the test. Each sample was compression molded into a disk of a 25 mm diameter and 1 mm thickness, and the measurement was done using this sample sandwiched in the parallel plates.

The DC electrical conductivity of the compression molded samples of dimensions 22 mm × 1 mm × 1 mm were measured under an applied voltage of 90 V using a Loresta GP resistivity meter (MCP-T610 model, Mitsubishi Chemical Co., Tokyo, Japan), connected with an ESP four-pin probe to eliminate the influence of contact resistance. Three molded samples were analyzed for each blend composite system, and three readings were taken from each sample face; the results were averaged, and the average was recorded as the electrical conductivity.

The mechanical testing for composites was done using an Instron tensile tester (Model no 5965-Norwood, MA, USA) at a crosshead speed of 50 mm/min. Four molded samples prepared according to ASTM D638 type IV specifications were tested and the results were averaged for each blend system.

## 3. Results

### 3.1. Blend Morphology

**Figure 1:**
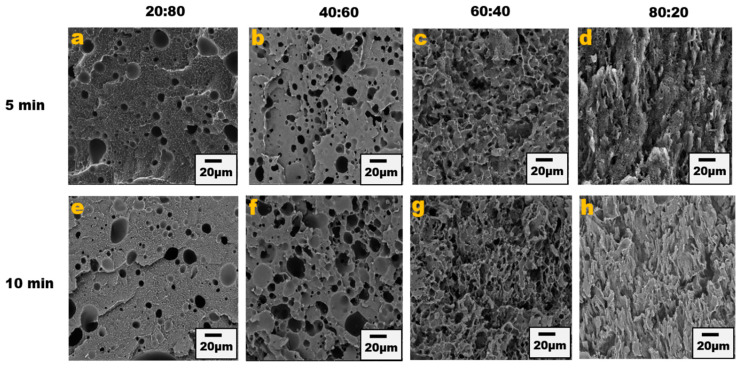
Blend morphology of PPE/HDPE/1 wt% CNT. Top images from (**a**–**d**) are 5 min mixing time with increasing PPE compositions; bottom images from (**e**–**h**) are 10 min mixing time.

**Figure 2:**
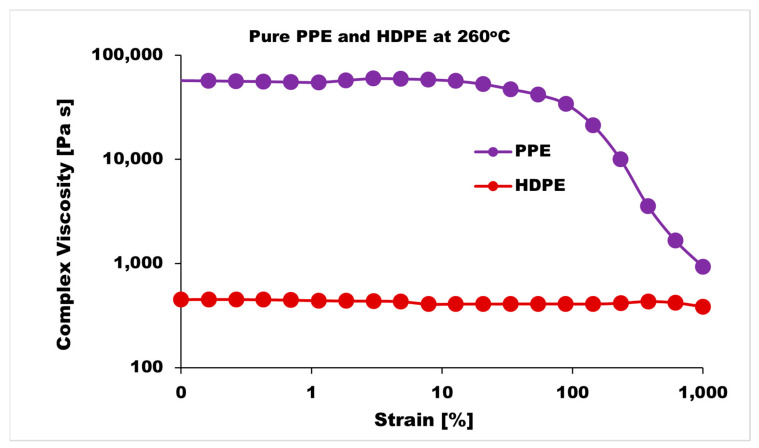
Strain sweeps of pure PPE and HDPE polymers at 260 °C.

**Figure 3:**
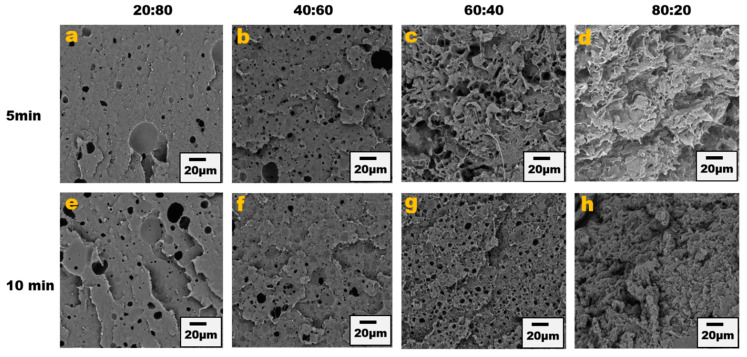
Blend morphology of PPE/HDPE/1 wt% CNT/ 2 wt% SEBS. Top images from (**a**–**d**) are 5 min mixing time with increasing PPE compositions; bottom images from (**e**–**h**) are 10 min mixing time.

The blend morphology was studied by etching the PPE phase with chloroform solvent; this provided micrographs that showed that by increasing the PPE concentration in the blend, the blend structure changes from a dispersed morphology to a co-continuous morphology. Figure 1 shows the different morphologies at a different blend composition with 1 wt% CNT for 5 min and 10 min of mixing. The images show approximately the same morphological changes for the two different mixing times, though a more irregular and deformed droplet domain was evident at 10 min of mixing for the PPE/HDPE/40:60 blend composition in Figure 1f. With CNT pre-localized in the HDPE matrix phase, the force exerted from the CNT filler during processing was countered by the high viscosity of the PPE droplet phase, leading to breakup and irregular domain sizes (Figure S1). In addition, owing to the high viscosity of the PPE phase (shown in Figure 2), the droplets breakup and transition to co-continuous morphology as the increase in the PPE concentration is delayed. This is particularly true in the absence of any interphase modifier (SEBS), as shown in Figure 3c,g. At 60% PPE, the co-continuous morphology is not pronounced. Though the shear forces acting on the droplets result in their deformation, these forces are countered by the restorative forces, such as the droplet elasticity and interfacial tension between the phases [35]. Conversely, the addition of the SEBS triblock copolymer results in a substantial decrease in the droplet size when comparing the morphologies of Figure 1 and Figure 3 for all the blend compositions at both mixing times.

The presence of the copolymer connects more with the PPE phase as it is miscible with styrene segments of the copolymer; thus, there is more migration of the copolymer to the interphase, bridging between the PPE and HDPE, hence the reduction in the droplet size and stabilization of the morphology shown in the analysis in Figure S2. The solvent extraction of PPE was carried out on both the uncompatibilized and compatibilized blend system and the result shows that PPE has a higher tendency to percolate in the HDPE matrix in the presence of the SEBS copolymer (Figure S3).

### 3.2. Electrical Conductivity

**Figure 4:**
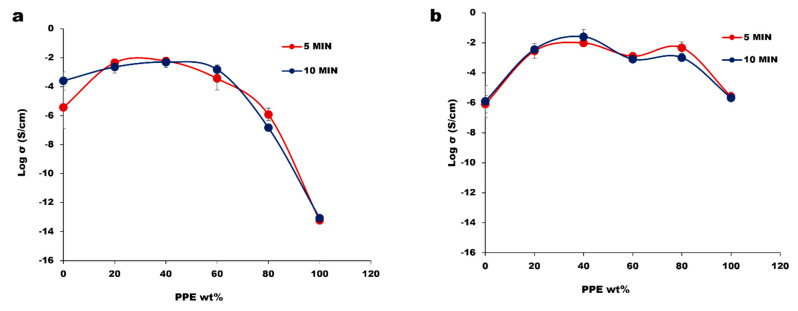
The electrical conductivity of polymer blend nanocomposites at different mixing time of 5 and 10 min: (**a**) PPE/HDPE/1 wt% CNT; (**b**) PPE/HDPE/1 wt% CNT/2 wt% SEB.

The establishment of a conductive pathway is one paramount factor for achieving high electrical conductivity, and the conductive pathway corresponds to a CNT network structure within the matrix. To achieve this, both an optimum dispersion and distribution are important, i.e., not necessarily the greatest amount of dispersion and distribution but an optimum level. In a blend system, polymer–filler interaction affects the extent of filler dispersion [36], and the level of each constituent polymer’s affinity towards CNT is governed by thermodynamic factors. However, the system can be tuned in such a way that the CNT migrates to the targeted phase in the blend composites. The blend morphologies induced by kinetics in our system resulted in an improvement in the electrical conductivity in Figure 4.

**Figure 5:**
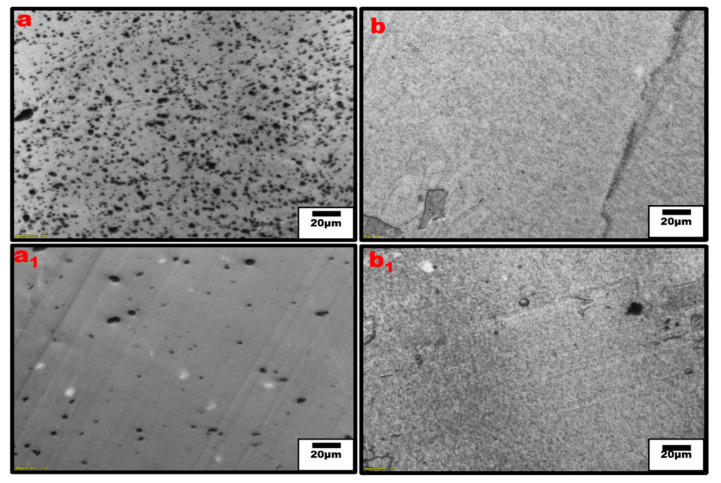
Optical micrograph of single polymer nanocomposites, (**a**,**a_1_**) PPE/CNT and PPE/CNT/SEBS, respectively, (**b**,**b_1_**) HDPE/CNT and HDPE/CNT/SEBS, respectively.

From our result, the impact of the mixing time between 5 and 10 min is minor, as the morphology and electrical properties follow the same variations as the blend concentration. Based on the rule of mixtures, we would expect the electrical conductivity to steadily decrease with the increase in the PPE content. By having CNT pre-localized in the HDPE phase, the CNT can migrate to the interface of PPE/HDPE blends and create percolated structures that are stronger than the pure HDPE nanocomposite. At a very high concentration of PPE, we observe a low electrical conductivity due to the lack of CNT network structure in the composites. The optical microscopy images in Figure 5 show large CNT agglomerates in the pure PPE nanocomposite (Figure 5a). This is believed to be due to the high viscosity of the polymer which limits CNT migration and dispersion; hence, we see a high amount of nanofiller agglomeration. On the other hand, the addition of a 2 wt% SEBS (2 wt% was selected as the optimum from the results shown in Figure S4) compatibilizer in Figure 5(a_1_,b_1_) shows a better CNT dispersion in the polymers PPE and HDPE, respectively. This relates to a substantial increase in conductivity in the blend system of approximately four orders of magnitude in Figure 4b. This can be explained by using Figure 5(a_1_), where we see a very different dispersion of CNT compared to Figure 5a for the same sample; that is, we do not have a homogenous distribution of CNT in this nanocomposite in the absence of a compatibilizer. Comparing the “a” image to the “a_1_” image, shows a significant reduction in the CNT agglomeration with the addition of the SEBS compatibilizer.

**Figure 6:**
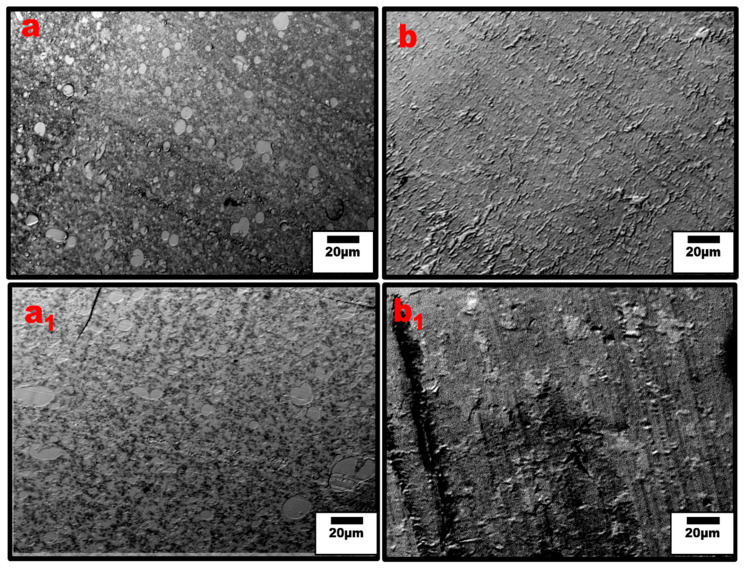
Optical micrographs of un-compatibilized (**a**) 20:80, (**b**) 80:20 PPE/HDPE, and compatibilized (**a_1_**) 20:80 and (**b_1_**) 80:20 PPE/HDPE blend nanocomposites. The dark spots are the CNT agglomerates. The matrix transparency is an indication of the level of CNT dispersion outside the agglomerates.

**Figure 7:**
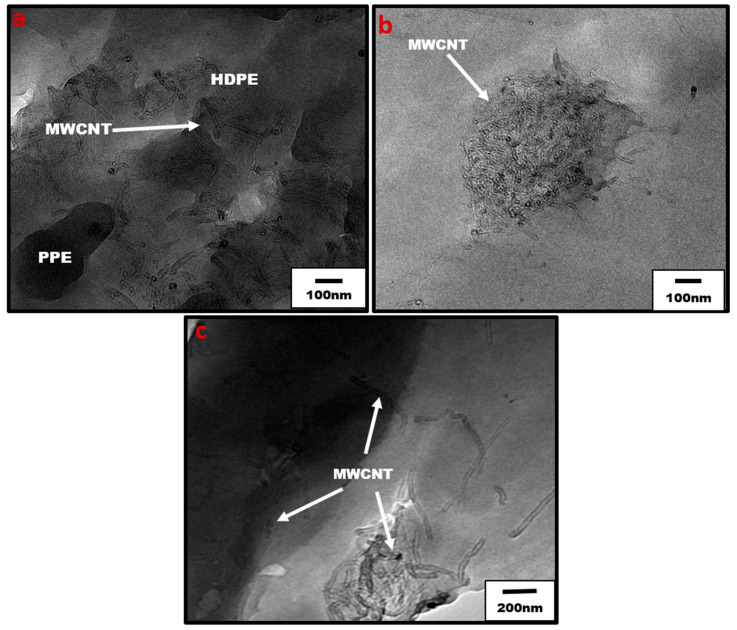
TEM of blend nanocomposites (**a**) uncompatibilized 20:80 PPE/HDPE with 1 wt% CNT, showing localization of CNT in HDPE (**b**) uncompatibilized 80:20 PPE/HDPE with 1 wt% CNT, and (**c**) compatibilized 80:20 PPE/HDPE blend nanocomposites with 1 wt% CNT. The arrows show the locations of CNT nanofiller.

Moreover, the extent of the CNT network structure in the polymer matrix is limited by a poor distribution and insufficient interfacial interaction between the nanofillers and polymer interface [37,38]. The addition of SEBS in our system shows an improved CNT dispersion and distribution leading to the significant increase in conductivity even for the PPE nanocomposite. However, it should be noted that some CNT agglomeration can be useful for electrical and mechanical properties. For example, they can be tuned in such a way as to achieve a systematic non-homogenous distribution, resulting in the formation of segregated structures, which are needed to improve the mechanical properties of CNT-reinforced polymer composites [34,35]. In Figure 6, optical micrographs for the different blend compositions are shown at low and high PPE concentrations. The optical densities of constitute phases can be used to differentiate them, i.e., based on the refractive index, the polymer with a higher refractive index is the darker phase. Figure 6a shows that for the 20:80 PPE/HDPE blend system, i.e., at a low PPE concentration, the PPE droplets show some detachment from the HDPE matrix as result of a lack of miscibility between the two polymers. However, the CNT did not migrate to the PPE phase; the filler remains localized in the HDPE matrix phase. With the addition of SEBS in Figure 6(a_1_), PPE droplets break into smaller droplets, increasing the interfaces, while the CNT filler shows some redistribution, and we can see CNT has migrated to the PPE phase. This is also shown in the TEM image in Figure 7a. Even though PPE is the thermodynamically preferred phase based on the wettability calculation using Young’s equation [39], see File S1 of the Supplementary Materials, we still have CNT localized in the HDPE phase. The interfacial tensions were first calculated using the harmonic and geometric mean, developed by Wu et al. [40]; see Table S1 for the result. The surface energies of the polymers used at 260 °C are shown in Table 2.

**Figure 8:**
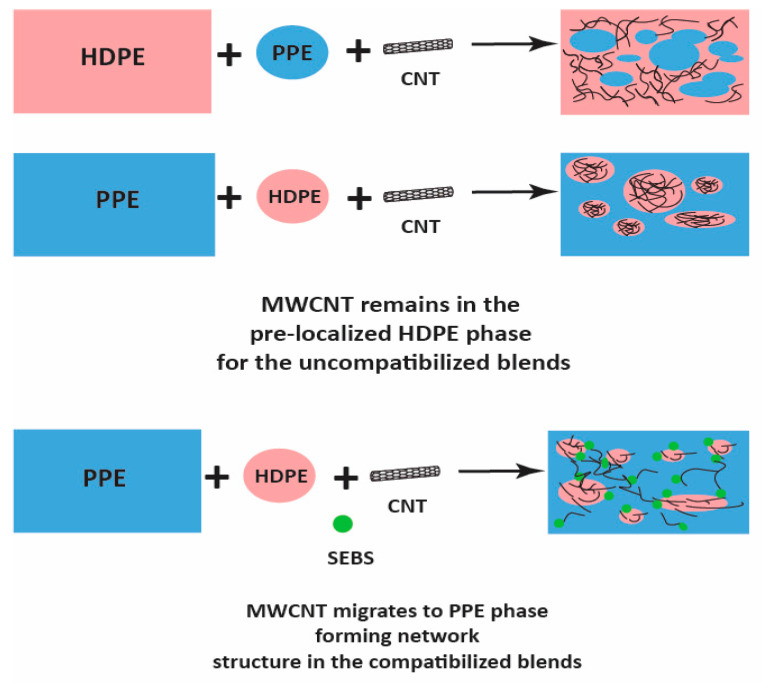
Schematic of MWCNT migration in the compatibilized blend nanocomposites.

Moreover, Figure 6b shows that at higher PPE concentration (80:20/PPE/HDPE), the HDPE minor phase percolates within the PPE matrix, and this agrees with the work of Wang et al. [43]. This shows the ability of the HDPE to form a continuous phase even at low concentrations. Nevertheless, the CNTs in the HDPE are not able to form a network structure, perhaps due to the highly stretched continuous HDPE phase, which leads to some gaps between the individual CNT filler. In addition, there were some CNT agglomerates formed (Figure 7b) that could reduce the level of percolation. However, with the addition of the SEBS triblock copolymer, the stretching of the HDPE phase was reduced and the triblock possibly also reduced the viscosity of the PPE phase, allowing for CNT migration, as shown in Figure 7c. The schematic in Figure 8 shows the mechanism of CNT movement in the uncompatibilized and compatibilized blends. This migration in the presence of the SEBS copolymer can be attributed to its ability to reduce the melt viscosity of the PPE polymer, allowing for CNT localization at the interphase and across the interphase into the PPE phase, as shown in the TEM image in Figure 7c.

### 3.3. Mechanical Properties of the Blend System

**Figure 9:**
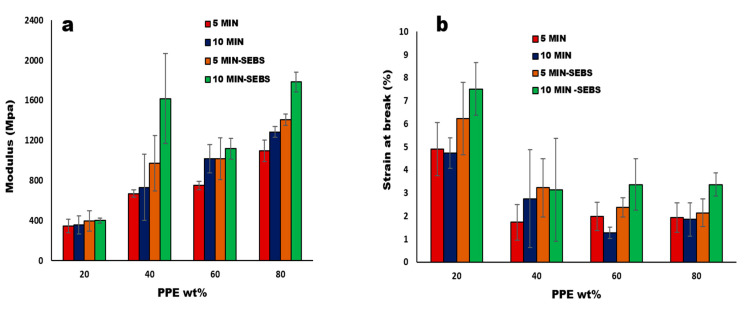
(**a**) Modulus and (**b**) the elongation at break, at increase in the weight% of PPE polymer in PPE/HDPE blend nanocomposites with 1 wt% MWCNT.

The major determining factor for the impact of CNT nanofillers in the polymer matrix is the bonding and strength of the interface, which correlates to the interfacial load transmission from the matrix to the filler surface [37]. In our system, the PPE polymer is more rigid and fragile, hence increasing the PPE concentration in the blends and increasing the modulus and tensile strength at the break (Figure S5). Consequently, in Figure 9, we see that the addition of the 2 wt% triblock copolymer in the blend nanocomposites shows an enhanced Young’s modulus for 80:20:1 /PPE/HDPE/CNT, with a 38.8% increase for 10 min mixing and a 28.5% increase for 5 min of mixing time. The higher increase at a higher mixing time is attributed to the longer shearing time, allowing for a better CNT dispersion and less agglomeration. This agrees with a previous work in the literature [44] which showed that a longer mixing time can result in a better interfacial adhesion and an improved interfacial interaction. This result confirms that the CNT migration occurs with the addition of the SEBS copolymer, as shown in Figure 7c. In the same way, the elongation at the break shown in Figure 9b is improved at 10 min of mixing time for the system with a low concentration of PPE polymer, whereas when the PPE concentration increases, we see a general reduction in elongation. However, at a higher PPE concentration at 60:40 and 80:20 PPE/HDPE, the addition of SEBS helps to improve the ductility of the blend nanocomposite, and we achieve a 164.6% and 82.2% increase in elongation, respectively, at 10 min of mixing time.

Furthermore, both the CNT nanofiller and the SEBS copolymer worked synergistically to improve the stiffness of our blend composites (see Figure S6), while the filler dispersion contributes to an effective load transfer. The copolymer plays a role in improving the miscibility in the multiphase system, hence the increase in the modulus and strength at the break seen for the compatibilized blend nanocomposites at 80:20 composition, as shown in Table 2. Therefore, the interfacial interaction in compatibilized PPE/HDPE/CNT composites correlates with the improvement in the tensile properties. In same way, the work conducted by Zhang and Sundararaj [45] with LLDE/PEMA and clay nanocomposites, in which they show that matching PEMA chemical structure with that of the LLDPE of their system, provides a better interaction between the clay filler and the matrix. Moreover, the 20:80 PPE/HDPE blend composition shows a higher ductility in the presence of the copolymer than without the copolymer, namely, by about a 340% increase in the elongation at the break. This shows that the addition of the CNT nanofiller generally improves the Young’s modulus and tensile strength at the break but not the ductility (see Table 3).

## 4. Conclusions

In this work, we showed the interplay between the blend morphology, compatibilization process, and the target blend property using kinetic factors to control the CNT migration. We achieved this by systematically selecting a high temperature, high viscosity PPE, which has a good dimensional stability and low viscosity HDPE polyolefin, which helps improve PPE processability. We showed that the addition of SEBS triblock copolymer significantly reduced the droplet domain size in the dispersed blend morphology, confirming SEBS as a good compatibilization agent for the PPE/HDPE blend system. At a high PPE content greater than 60%, where PPE is the matrix, increasing the PPE concentration results in a low electrical conductivity. This is likely a result of CNT agglomeration within the PPE matrix because of PPEs high viscosity. This is particularly evident at the 80:20/PPE/HDPE blend. However, the addition of the SEBS compatibilizer reduces the viscosity, allowing for more CNT migration and network formation, and hence a significant increase in conductivity of about four orders of magnitude compared to the uncompatibilized blend. In the same way, the tensile property of our system shows an improvement in the compatibilized blend nanocomposites due to the tuned interaction and connections between the CNT filler and the SEBS compatibilizer. Nevertheless, the ductility is improved more in the presence of the compatibilizer up to substantial amount of a 339.8% increase than in the CNT-reinforced polymer nanocomposites.

## Figures and Tables

**Table 1:** Blend compositions and processing conditions.

Samples	Blend Compositions	Filler	Copolymer	Mixing Time
HDPE-MB	0:100	10 wt%	-	-
PPE/HDPE/CNT	80:20, 20:80	1 wt%	-	5, 10 min
PPE/HDPE/CNT/SEBS	20:80, 40:60, 60:40, 80:20	1 wt%	1, 2, 3 wt%	5, 10 min
Pure PPE/HDPE	20:80, 80:20	-	-	5, 10 min
PPE/HDPE	20:80, 80:20	-	2	5, 10 min

**Table 2:** Dispersive and polar components of and total surface tension for PPE, HDPE, and MWCNT at 260 °C.

Materials	Total Surface Tension (mN/m) $\gamma_{T}$	Dispersive Surface Tension (mN/m) $\gamma_{d}$	Polar Surface Tension (mN/m) $\gamma_{p}$	References
PPE	28.4	22.2	6.2	[41,42]
HDPE	22	22	0	[43]
MWCNTs	27.8	17.6	10.2	[41]

**Table 3:** Tensile properties of the different blend samples at 80:20 and 20:80 composition.

Samples	Tensile Strength at Break (MPa)	Young’s Modulus (MPa)	Elongation at Break (%)
PPE/HDPE 80/20	15.40 ± 0.7	1029.45 ± 24.7	3.00 ± 1.9
PPE/HDPE/SEBS/80/20/2	28.30 ± 4.9	1557.18 ± 119.0	2.90 ± 0.3
PPE/HDPE/CNT/80/20/1	21.25 ± 2.2	1285.70 ± 54.2	3.46 ± 0.7
PPE/HDPE/SEBS/CNT/80/20/2/1	38.27± 0.2	1784.26 ± 99.9	5.97 ± 0.5
PPE/HDPE 20/80	11.49 ± 1.9	210.4 ± 36.4	15.27 ± 3.4
PPE/HDPE/SEBS/20/80/2	15.40 ± 0.6	275.06 ± 20.6	25.2 ± 2.6
PPE/HDPE/CNT/20/80/1	14.05 ± 2.9	356.04 ± 92.3	5.73 ± 0.7
PPE/HDPE/SEBS/CNT/20/80/2/1	16.86 ± 0.6	399.39 ± 27.1	9.51 ± 1.1

## Data Availability

The data presented in this work are available upon request from the corresponding author.

## Supplementary Materials

The following supporting information can be downloaded at: https://www.mdpi.com/article/10.3390/nano13061039/s1, Figure S1: PPE/HDPE/20:80/ 1 wt% CNT at 10 min; Figure S2: Domain sizes of PPE with and without SEBS; Figure S3: Solvent extraction of PPE using chloroform; Figure S4: Electrical conductivity at different copolymer composition and different blend composition; Figure S5: Strength at break and the elongation at yield of the polymer blend nanocomposites at 1 wt% CNT; Figure S6: (a) modulus, (b) strength at break, (c) elongation at break, and (d) elongation at yield of the different polymer blend samples at 80:20 and 20:80 composition; File S1: The Young’s equation [39] for calculation of wettability parameter (ω) in PPE/HDPE blend; Table S1: Geometric and harmonic mean values of PPE/HDPE, PPE/MWCNT, and HDPE/MWCNT.
