# Supplementary material for: Carbon Nanotube Migration in a Compatibilized Blend System, Leading to Kinetically Induced Enhancement in Electrical Conductivity and Mechanical Properties
Source: Nanomaterials (Basel). 2023 Mar 14;13(6):1039. doi: 10.3390/nano13061039 (PMC10051242; doi:10.3390/nano13061039)
Supplement: Supplementary file 1 [file nanomaterials-13-01039-s001.zip › nanomaterials-2229690-SI.pdf]

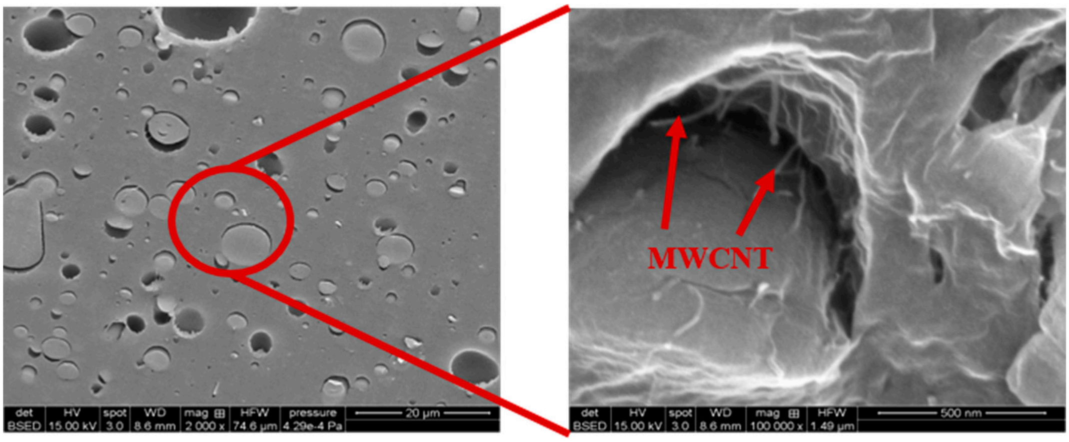

Figure S1. - PPE/HDPE/20:80/ 1 wt% CNT at 10min.

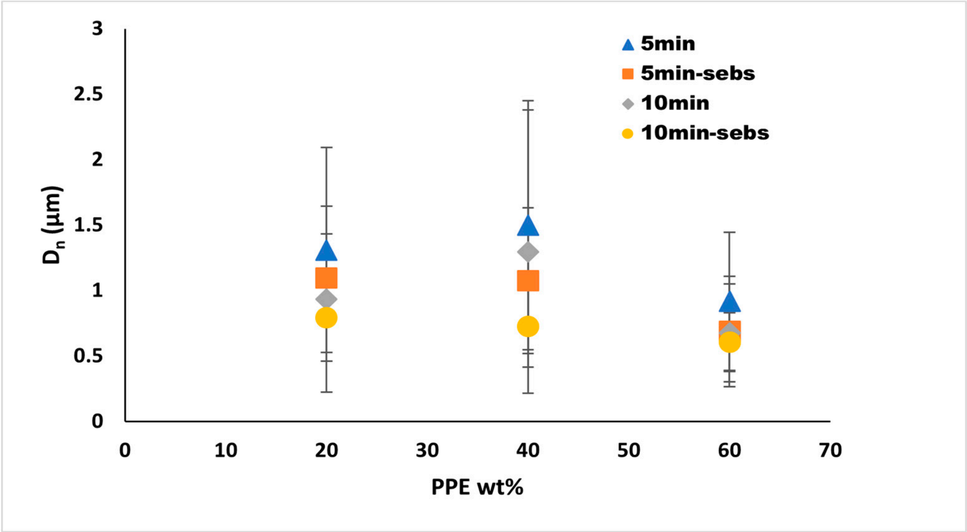

Figure S2. – Domain sizes of PPE, with and without SEBS.

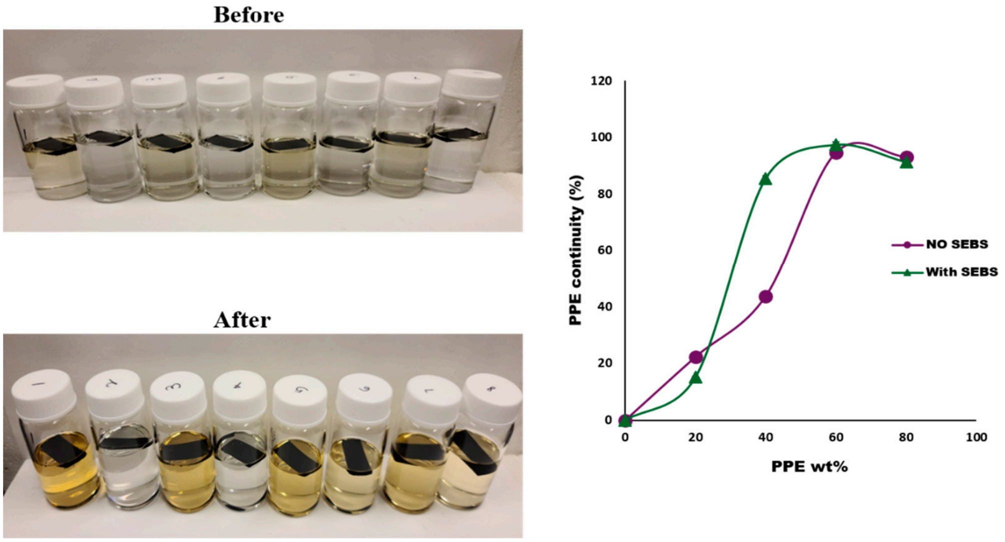

Figure S3 - Solvent extraction of PPE using chloroform.

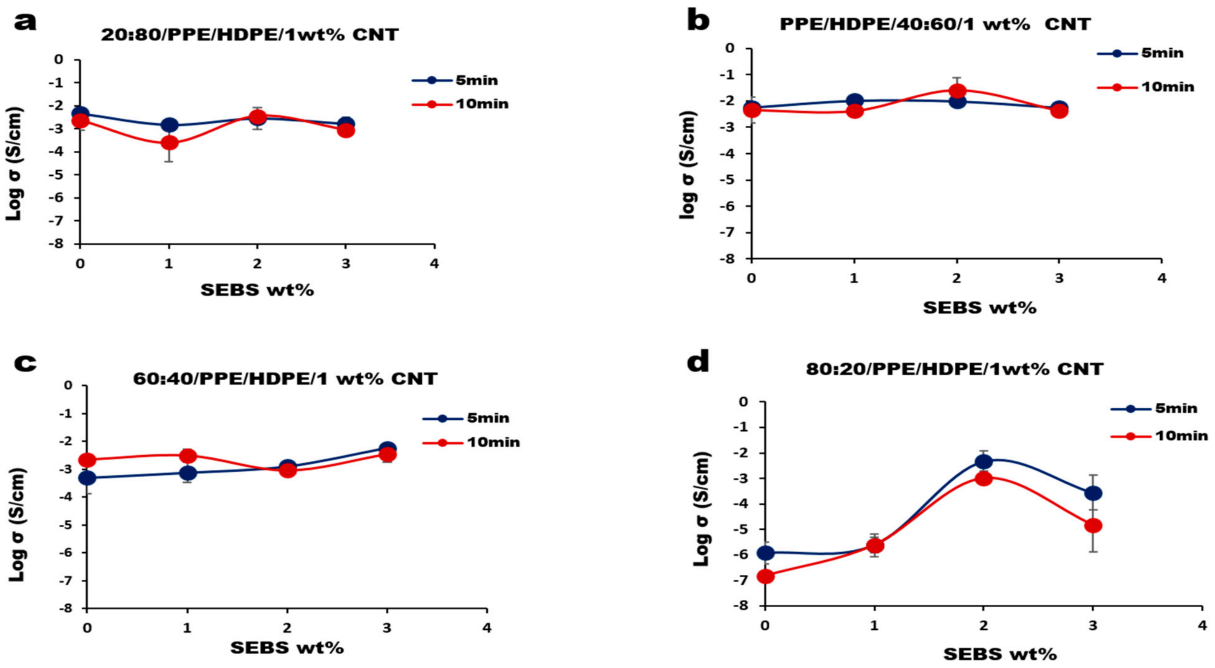

Figure S4 - Electrical conductivity at different Copolymer composition and different blend composition

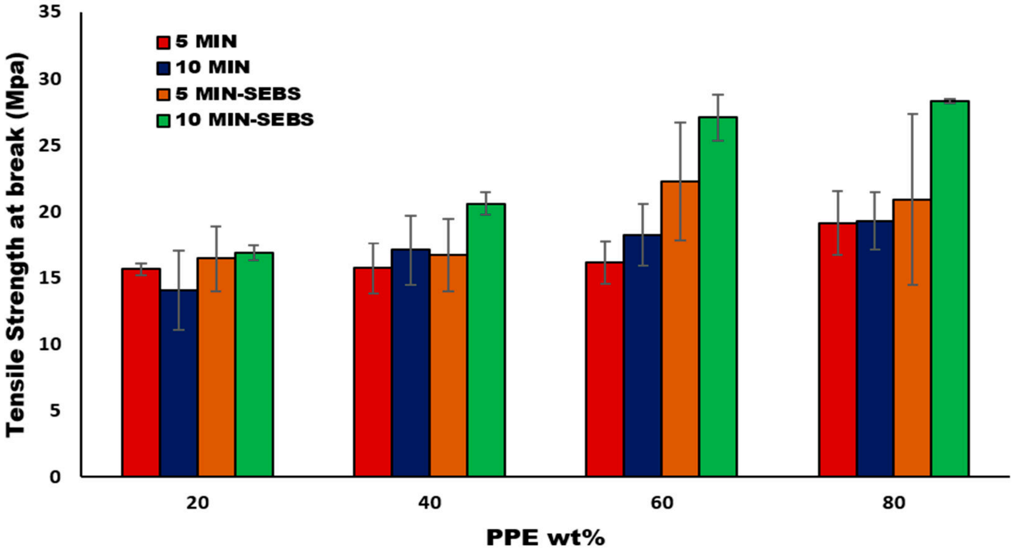

Figure S5. Strength at break of the polymer blend nanocomposites at 1wt% CNT

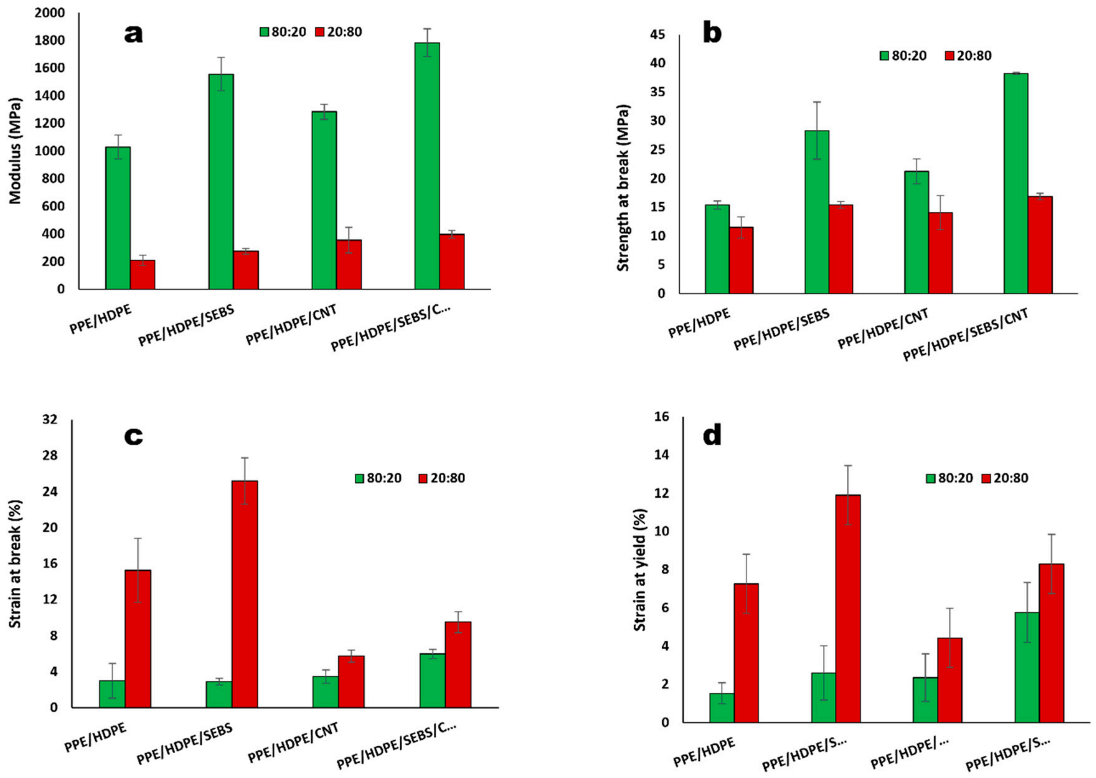

Figure S6. a) Modulus, b) Strength at break, c) elongation at break and d) elongation at yield of the different polymer blend samples at 80:20 and 20:80 composition.

File S1. The Young's equation [1] for calculation of wettability parameter (ω) in PPE/HDPE blend:

$$\boldsymbol{\omega_{AB}} = \frac{\gamma_{polymerB-filler} - \gamma_{polymerA-filler}}{\gamma_{polymerA-polymerB}} \quad \text{Eq. S1}$$

$\omega_{AB} > 1$ Filler localizes within polymer A

$\omega_{AB} < -1$ Filler is within polymer B.

$-1 < \omega_{AB} < 1$ Filler distributes at the interface.

The geometric and harmonic mean developed by Wu [2]

$$\gamma_{AB} = \gamma_A + \gamma_B - 2\left[\sqrt{\gamma_A^d\,\gamma_B^d} + \sqrt{\gamma_A^p\,\gamma_B^p}\right] \quad \text{Eq. S2}$$

$$\gamma_{AB} = \gamma_A + \gamma_B - 4\left[\frac{\gamma_A^d\,\gamma_B^d}{\gamma_A^d+\gamma_B^d} + \frac{\gamma_A^p\,\gamma_B^p}{\gamma_A^p+\gamma_B^p}\right. \quad \text{Eq. S3}$$

Table S1. Geometric and Harmonic mean values of PPE/HDPE, PPE/MWCNT and HDPE/MWCNT

Interfacial tensions	Geometric	Harmonic
$\boldsymbol{\gamma_{PPE/HDPE}}$	**6.2**	**6.2**
$\boldsymbol{\gamma_{PPE/MWCNT}}$	**0.76**	**1.51**
$\boldsymbol{\gamma_{HDPE/MWCNT}}$	**10.45**	**10.69**
**wettability**	**1.56**	**1.48**

Reference

[1] M. Sumita, K. Sakata, S. Asai, K. Miyasaka, and H. Nakagawa, "Dispersion of fillers and the electrical conductivity of polymer blends filled with carbon black," *Polym. Bull.*, vol. 25, no. 2, pp. 265–271, 1991, doi: 10.1007/BF00310802.

[2] S. Wu, "Calculation of interfacial tension in polymer systems," *J. Polym. Sci. Part C Polym. Symp.*, vol. 34, no. 1, pp. 19–30, 2007, doi: 10.1002/polc.5070340105.
